# Kitchen and Stores Department: "Tip and Non-Tip" Kitchen Boilers

**Published:** 1915-12-25

**Authors:** 


### Kitchen and Stores Department: "Tip and Non-Tip" Kitchen Boilers.

*To the Editor of* The Hospital.

Sir,—With reference to your article, December 18, on the "Educational Value of King's College Hospital," you criticise in the paragraph "The Kitchen and Stores Department" the installation of the non-tip pattern boiling coppers. We think it due to ourselves to mention that in our original proposals the advantages of the "tip" pattern or "movable" boiling copper were pointed out to the committee, and it was this form of copper which was included for in our first scheme. For reasons unknown to us, however, the non-tip pattern was eventually decided upon, much to our regret.

The writer of your article may not be aware that in the principal hospitals in this country where our apparatus are in use the "movable" or "tip" pattern copper is employed, and that this copper was designed by us some twenty years ago especially for hospital work. It is hard, under these circumstances, to read a criticism which implies a want of knowledge and experience on our part. We ought to say, perhaps, that with the non-tip coppers installed care has been taken to construct the inner pans in such a manner as to ensure all liquid draining away through the fullway and easily cleaned draw-off cock.

Hoping that you can see your way to inserting this letter of explanation in your Journal,—We are, your obedient servants,

For James Slater and Co. (Engineers), Ltd.,
C. H. V.

50 and 51 Wells Street, Oxford Street, W.
December 21, 1915.

[We welcome this useful letter. The writer of the article has known and approved James Slater's fittings from the earliest days. He was informed, when he raised the point of the boilers referred to, and expressed regret that Messrs. Slater's specially designed movable boilers had not been used, that the management of the firm had changed, and that the non-tip boiler was recommended against the views and wishes of some of the hospital staff.—Ed. The Hospital.]

